# Supplementary figures and images for: Calculating and comparing codon usage values in rare disease genes highlights codon clustering with disease-and tissue- specific hierarchy
Source: PLoS One. 2022 Mar 31;17(3):e0265469. doi: 10.1371/journal.pone.0265469 (PMC8970475; doi:10.1371/journal.pone.0265469)

A

## Homo S. Muscle

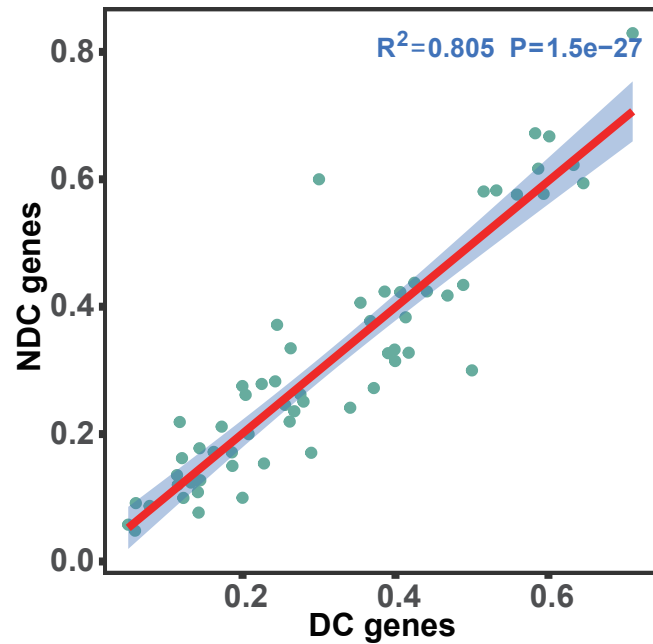

B

## Homo S. Skin

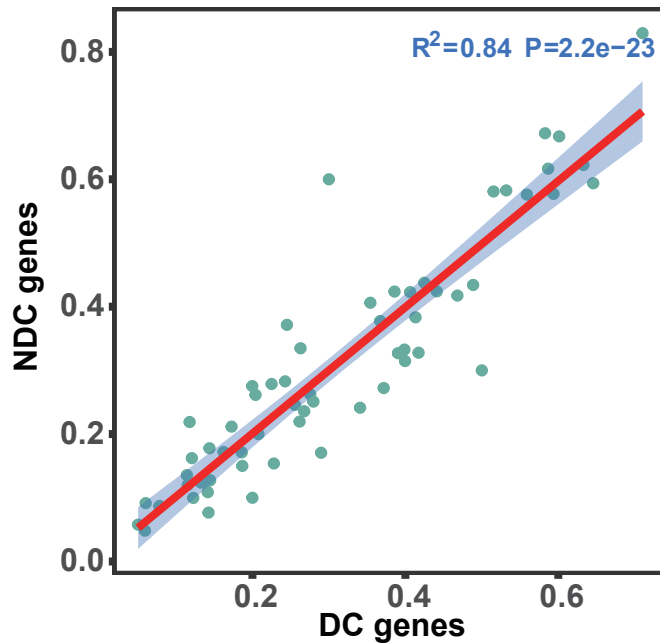

C

## Homo S. Kidney

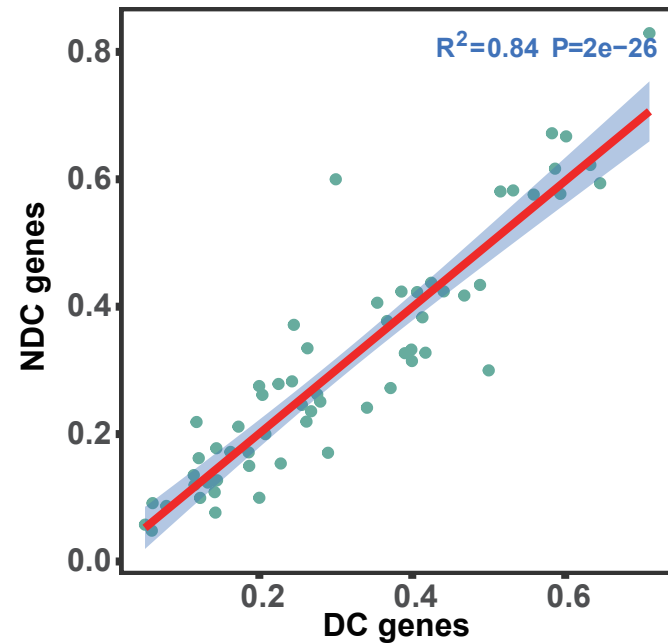

Supplement: S1 Fig — The test demonstrated that DC and NDC genes CU values correlate significantly in muscle, skin and kidney (p<0.05). (PDF) [file pone.0265469.s001.pdf]

High Expressed Genes

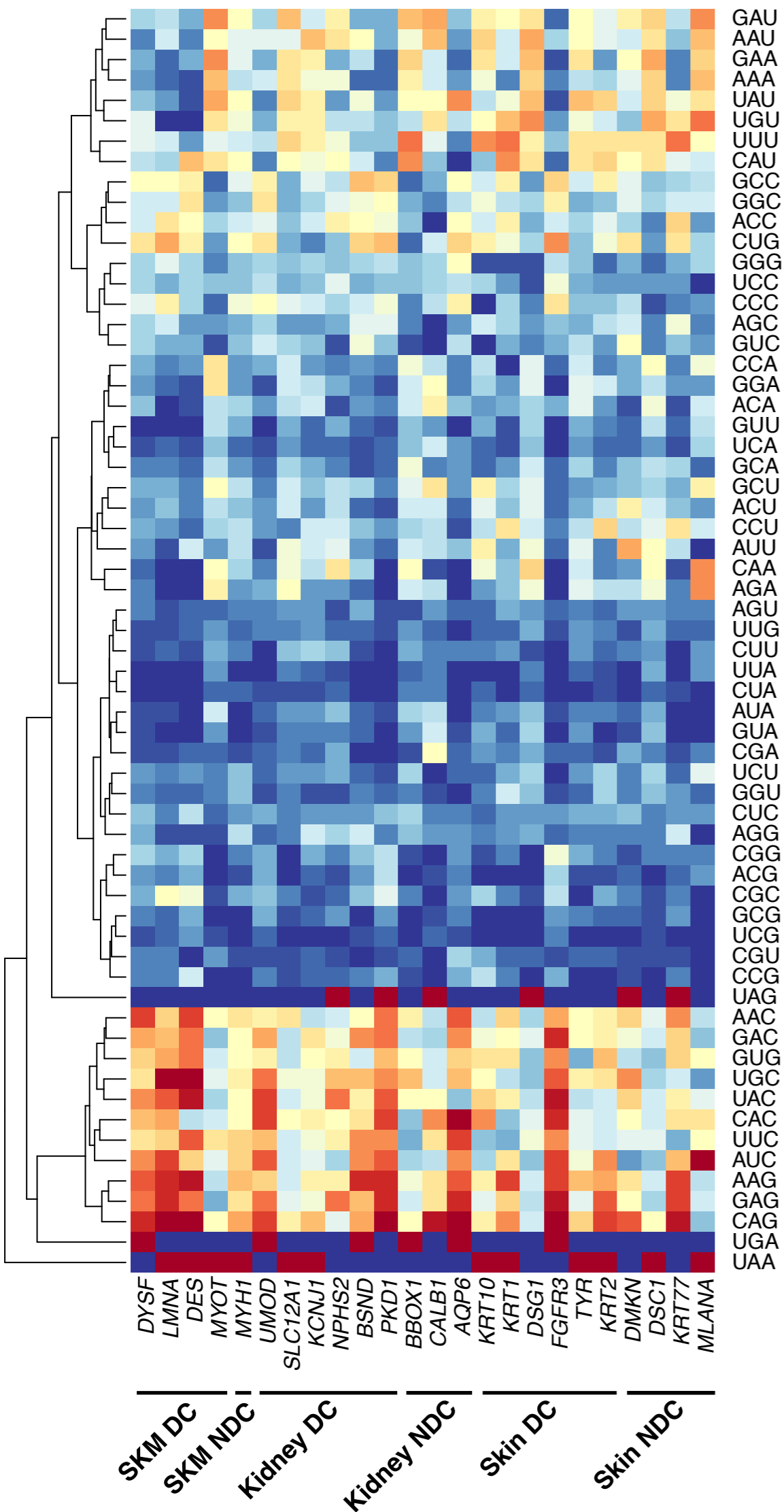

Medium Expressed Genes

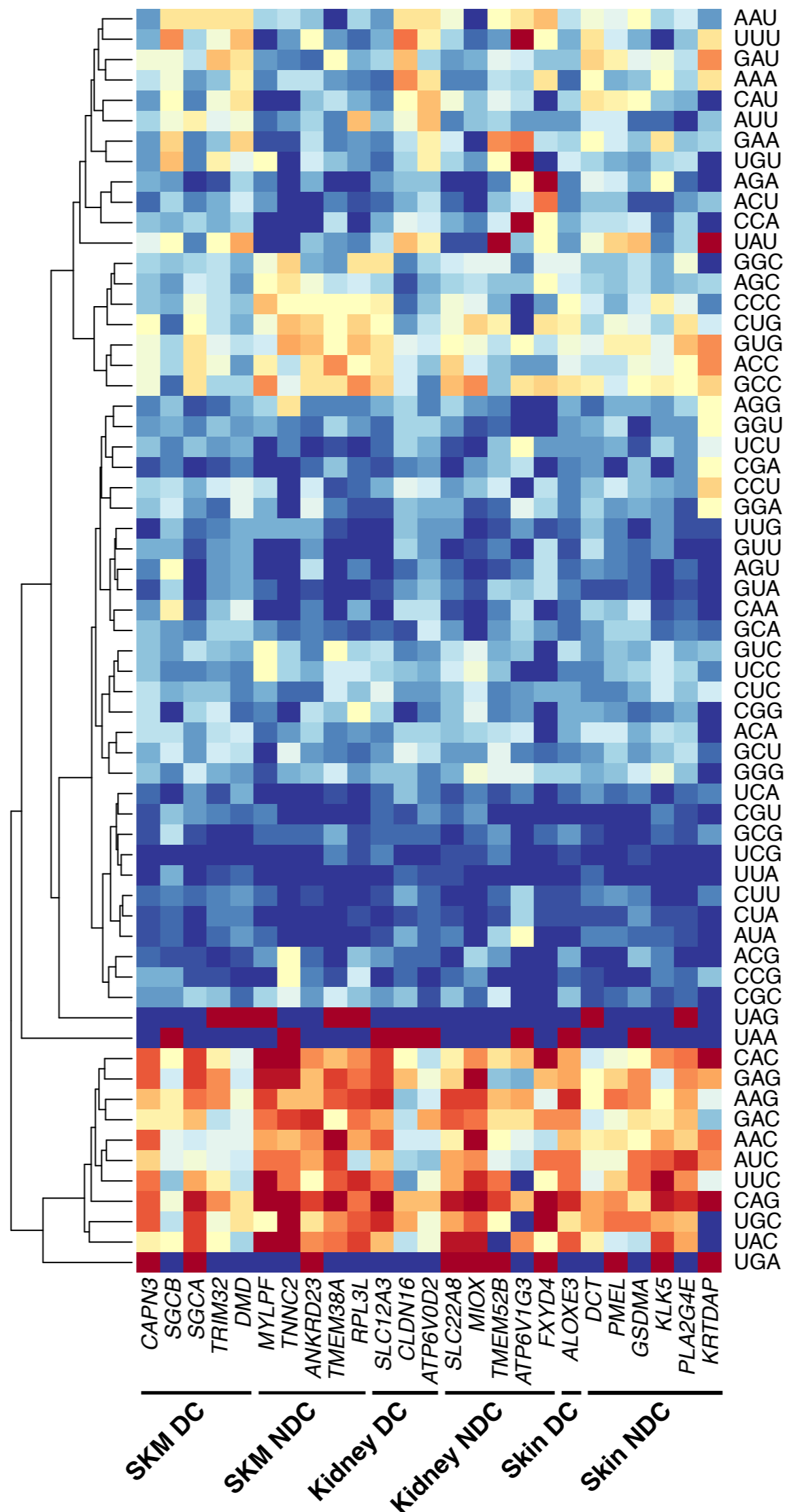

Low Expressed Genes

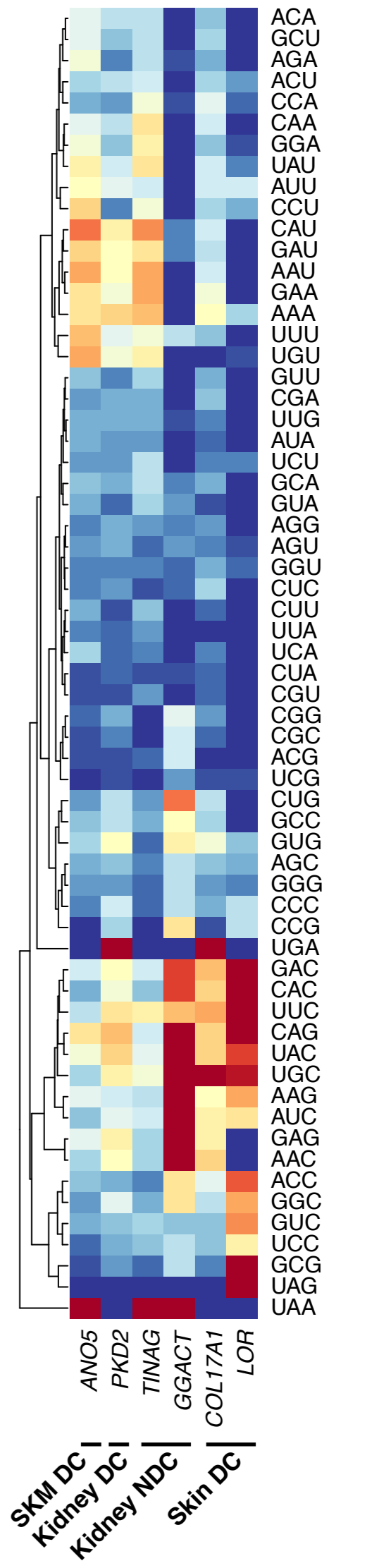

Supplement: S2 Fig — Rows were clustered based on Euclidean distance. The color coding varies from dark blue to red with low to high CU values respectively. CUB fingerprint and CU values among high-, medium- and low-expressed HSA genes. DC and NDC genes were considered depending on their expression level. CUB fingerprints have high similarity, meaning that indeed, grouping genes for their expression level yields a similar CU value trend. Codons AAC, GAC, UGC, UAC, CAC, UUC, AUC, AAG, GAG and CAG are more frequently used both in high- and medium-expressed genes while GUG is only present in highly expressed genes. Codons GAC, CAC, UUC, CAG, UAC, UGC, AAG, AUC, GAG, AAC, ACC, GGC, GUC, UCC and GCG are more frequently used in low-level expressed genes. A few codons have lower CU values such as UCC (Ser), ACC (Thr), GGC (Gly), GUC (Val) and GCG (Ala) in low-expressed genes. Some highly expressed DC genes have more codons with higher CU values, like DYS, LMNA and DES (muscle), UMOD and PKD1 (kidney) and FGFR3 (skin). In medium-expressed genes, the trend is opposite, with some NDC genes that show higher CU values like MLPF, TNNC2, TMEM3BA (muscle) and NCLZ2, MCX (kidney). Interestingly, UAA is the most used stop codon in highly expressed genes since it induces translation termination with higher speed and accuracy at the ribosomal level and can be read by both release factors eRF1 and eRF2 [31, 32]. UAG and UGA have a similar frequency in all tissue genes and expression levels. (PDF) [file pone.0265469.s002.pdf]
