# Supplementary material for: Calculating and comparing codon usage values in rare disease genes highlights codon clustering with disease-and tissue- specific hierarchy
Source: PLoS One. 2022 Mar 31;17(3):e0265469. doi: 10.1371/journal.pone.0265469 (PMC8970475; doi:10.1371/journal.pone.0265469)
Supplement: S1 Table — (DOCX) [file pone.0265469.s003.docx]

**Supplementary Table 1**

| **SEQUENCES REFERENCES** | **NCBI LINK** |
| --- | --- |
| **Rhinolophus ferrumequinum (Greater horseshoe bat )** | https://www.ncbi.nlm.nih.gov/assembly/GCA_004115265.3 |
| **Mus musculus** **(House mouse)** | https://www.ncbi.nlm.nih.gov/gene/13405 |
| **Felis catus (Cat)** | https://www.ncbi.nlm.nih.gov/assembly/GCF_000181335.3 |
| **Canis lupus familiaris (Dog)** | https://www.ncbi.nlm.nih.gov/assembly/GCF_000002285.3/ |
| **Equus caballus (Horse)** | https://www.ncbi.nlm.nih.gov/assembly/GCF_002863925.1 |
| **Bos taurus (Cattle)** | https://www.ncbi.nlm.nih.gov/assembly/GCF_002263795.1 |
| **Microcebus murinus (Gray mouse lemur)** | https://www.ncbi.nlm.nih.gov/nuccore/1135509992 |
| **Galeopterus variegatus (Sunda flying lemur )** | https://www.ncbi.nlm.nih.gov/nuccore/640470054 |
| **Callithrix jacchus (Common marmoset)** | https://www.ncbi.nlm.nih.gov/assembly/GCF_000004665.1/ |
| **Macaca mulatta (Rhesus macaque)** | https://www.ncbi.nlm.nih.gov/assembly/GCF_000772875.2/ |
| **Nomascus leucogenys (Northern white-cheeked gibbon)** | https://www.ncbi.nlm.nih.gov/nuccore/350542784 |
| **Pongo abelii (Sumatran orangutan)** | https://www.ncbi.nlm.nih.gov/nuccore/180204958 |
| **Gorilla gorilla (Western gorilla)** | https://www.ncbi.nlm.nih.gov/assembly/GCF_000151905.2/ |
| **Pan troglodytes (Chimpanzee)** | https://www.ncbi.nlm.nih.gov/assembly/GCF_000001515.7/ |
| **Homo sapiens (Human)** | Various genes see supp table 2, 3 and 4 |
