# Supplementary material for: Calculating and comparing codon usage values in rare disease genes highlights codon clustering with disease-and tissue- specific hierarchy
Source: PLoS One. 2022 Mar 31;17(3):e0265469. doi: 10.1371/journal.pone.0265469 (PMC8970475; doi:10.1371/journal.pone.0265469)
Supplement: S2 Table — In order to prioritize muscle genes, we selected those with higher expressions from the skeletal muscle-enriched genes list of the Human Protein Atlas database (https://www.proteinatlas.org/search/tissue_specificity_rna:skeletal%20muscle;Tissue%20enriched+AND+sort_by:tissue+specific+score+AND+show_columns:groupenriched). All data (RNA, TS, TPM, and Protein expression scores) were also obtained by the Human Protein Atlas database. *RNA TS TPM indicates RNA level reported as mean TPM (transcripts per million), in referred tissue, skeletal muscle in this case. **Protein expression scores are based on a best estimate of the "true" protein expression from a knowledge-based annotation in the selected tissue, skeletal muscle in this case. ***Tissue specificity is based on data found in the graph called “HPA tissue dataset”, a sub-category of the “RNA sample summary” section in the HPA site, for each gene. The RNA summary section shows normal distribution of individual samples across the datasets of multiple RNA-seq analyses visualized with box plots. “Only” is used for a gene transcript present only in the specific tissue (skeletal muscle). “Predominantly” is used when the majority of a gene transcript is present in the specific tissue (skeletal muscle). “All” is used for a gene transcript present in all tissues. (DOCX) [file pone.0265469.s004.docx]

**Supplementary TABLE 2: SKM genes**

1. **DISEASE CAUSING GENES**

|  | **GENE** | **NCBI LINK** | **RNA TS TPM*** | **PROTEIN EXPRESSION (score)**** | **OMIM NUMBER** | **TISSUE SPECIFICITY** |
| --- | --- | --- | --- | --- | --- | --- |
| 1 | DYSF: Homo sapiens dysferlin (DYSF), transcript variant 1, mRNA | https://www.ncbi.nlm.nih.gov/nuccore/NM_001130987.1 | 32,8 | High | 603009 | predominantly |
| 2 | CAPN3: Homo sapiens calpain 3 (CAPN3), transcript variant 1, mRNA | https://www.ncbi.nlm.nih.gov/nuccore/NM_000070.2 | 336,8 | Medium | 114240 | Predominantly |
| 3 | SGCB: Homo sapiens sarcoglycan beta (SGCB), mRNA | https://www.ncbi.nlm.nih.gov/nuccore/ NM_000232.4 | 35,4 | Medium | 600900 | Predominantly (the highest of two) |
| 4 | SGCA: Homo sapiens sarcoglycan alpha (SGCA), transcript variant 1, mRNA | https://www.ncbi.nlm.nih.gov/nuccore/NM_000023.3 | 181,4 | Medium | 600119 | Predominantly |
| 5 | LMNA: Homo sapiens lamin A/C (LMNA), transcript variant 1, mRNA | https://www.ncbi.nlm.nih.gov/nuccore/NM_170707.3 | 55 | High | 150330 | All |
| 6 | DES: Homo sapiens desmin (DES), mRNA | https://www.ncbi.nlm.nih.gov/nuccore/ NM_001927.3 | 5462 | High | 125660 | Only |
| 7 | MYOT: Homo sapiens myotilin (MYOT), transcript variant 1, mRNA | https://www.ncbi.nlm.nih.gov/nuccore/NM_006790.2 | 766,8 | High | 604103 | Only |
| 8 | ANO5: Homo sapiens anoctamin 5 (ANO5), transcript variant 1, mRNA | https://www.ncbi.nlm.nih.gov/nuccore/NM_213599.2 | 23,6 | Low | 608662 | One of the five |
| 9 | COL6A1: Homo sapiens collagen type VI alpha 1 chain (COL6A1), mRNA | https://www.ncbi.nlm.nih.gov/nuccore/ NM_001848.2 | 29,1 | Only smooth muscle | 120220 | Predominantly |
| 10 | TRIM32: Homo sapiens tripartite motif containing 32 (TRIM32), transcript variant 1, mRNA | https://www.ncbi.nlm.nih.gov/nuccore/NM_012210. | 5,4 | Medium | 602290 | All |
| 11 | **DMD**: Homo sapiens dystrophin (DMD), transcript variant Dp427m, mRNA | https://www.ncbi.nlm.nih.gov/nuccore/NM_004006.2 | 34,8 | Medium | 300377 | Only |

1. **NOT CAUSING DISEASES GENES**

|  | **GENE** | **NCBI LINK** | **RNA TS TPM*** | **PROTEIN EXPRESSION (score)**** | **TISSUE SPECIFICITY ***** |
| --- | --- | --- | --- | --- | --- |
| **1** | **ACTN3**: [Homo sapiens actinin alpha 3 (gene/pseudogene) (ACTN3), transcript variant 1, coding, mRNA](https://www.ncbi.nlm.nih.gov/nuccore/NM_001104.3) | <https://www.ncbi.nlm.nih>. gov/nuccore/NM_001104.3 | 556,5 | Not performed | Only |
| **2** | **MYLPF**: [Homo sapiens myosin light chain, phosphorylatable, fast skeletal muscle (MYLPF), transcript variant 1, mRNA](https://www.ncbi.nlm.nih.gov/nuccore/NM_013292.4) | <https://www.ncbi.nlm.nih>. gov/nuccore/NM_013292.4 | 6541,1 | Medium | Predominantly (the highest of three) |
| **3** | **TNNC2**: [Homo sapiens troponin C2, fast skeletal type (TNNC2), mRNA](https://www.ncbi.nlm.nih.gov/nuccore/NM_003279.2) | <https://www.ncbi.nlm.nih>. gov/nuccore/NM_003279.2 | 9898,9 | Medium | Only |
| 4 | ANKRD23: Homo sapiens ankyrin repeat domain 23 (ANKRD23), mRNA | <https://www.ncbi.nlm.nih>. gov/nuccore/NM_144994.7 | 495,5 | Medium | Predominantly (the highest of two) |
| 5 | LBX1: Homo sapiens ladybird homeobox 1 (LBX1), mRNA | <https://www.ncbi.nlm.nih>. gov/nuccore/NM_006562.4 | 9,1 | Not performed | Only |
| 6 | LSMEM1: Homo sapiens leucine rich single-pass membrane protein 1 (LSMEM1), mRNA | <https://www.ncbi.nlm.nih>. gov/nuccore/NM_182597 | 31,8 | Not performed | Predominantly |
| 7 | TMEM38A: Homo sapiens transmembrane protein 38A (TMEM38A), mRNA | <https://www.ncbi.nlm.nih>. gov/nuccore/NM_024074.2 | 199,7 | Medium | One of two |
| 8 | RPL3L: Homo sapiens ribosomal protein L3 like (RPL3L), mRNA | <https://www.ncbi.nlm.nih>. gov/nuccore/NM_005061.2 | 323,9 | Medium | Only |
| 9 | MYH1: Homo sapiens myosin heavy chain 1 (MYH1), mRNA | <https://www.ncbi.nlm.nih>. gov/nuccore/NM_005963.3 | 2753,9 | High | Only |
