# Supplementary material for: Calculating and comparing codon usage values in rare disease genes highlights codon clustering with disease-and tissue- specific hierarchy
Source: PLoS One. 2022 Mar 31;17(3):e0265469. doi: 10.1371/journal.pone.0265469 (PMC8970475; doi:10.1371/journal.pone.0265469)
Supplement: S3 Table — In order to prioritize skin genes, we selected those with higher expressions from the skin-enriched genes list of the Human Protein Atlas database (https://www.proteinatlas.org/search/tissue_specificity_rna:skin;Tissue%20enriched+AND+sort_by:tissue+specific+score+AND+show_columns:groupenriched). All data (RNA, TS, TPM, Protein expression scores and Tissue specificity) were also obtained by the Human Protein Atlas database. *RNA TS TPM indicates RNA level reported as mean TPM (transcripts per million), in referred tissue, skin in this case. **Protein expression scores are based on a best estimate of the "true" protein expression from a knowledge-based annotation in the selected tissue, skin in this case. ***Tissue specificity is based on data found in the graph called “HPA tissue dataset”, a sub-category of the “RNA sample summary” section in the HPA site, for each gene. The RNA summary section shows normal distribution of individual samples across the datasets of multiple RNA-seq analyses visualized with box plots. “Only” is used for a gene transcript present only in the specific tissue (skin). “Predominantly” is used when the majority of a gene transcript is present in the specific tissue (skin). “All” is used for a gene transcript present in all tissues. (DOCX) [file pone.0265469.s005.docx]

**Supplementary TABLE 3: Skin genes**

1. **DISEASE CAUSING GENES**

|  | **GENE** | **NCBI LINK** | **RNA TS TPM*** | **PROTEIN EXPRESSION (score)**** | **OMIM NUMBER** | **TISSUE SPECIFICITY** |
| --- | --- | --- | --- | --- | --- | --- |
| 1 | **KRT10**: Homo sapiens keratin 10 (KRT10), mRNA | https://www.ncbi.nlm.nih.gov/nuccore/NM_000421.3 | 18886 | High | 148080 | Only |
| 2 | **KRT1**: Homo sapiens keratin 1 (KRT1), mRNA | https://www.ncbi.nlm.nih.gov/nuccore/NM_006121.3 | 15454,7 | High | 139350 | Predominantly (one of three) |
| 3 | **DSG1**: Homo sapiens desmoglein 1 (DSG1), mRNA | https://www.ncbi.nlm.nih.gov/nuccore/NM_001942.3 | 725,7 | High | 125670 | Predominantly |
| 4 | ALOXE3: Homo sapiens arachidonate lipoxygenase 3 (ALOXE3), transcript variant 1, mRNA | https://www.ncbi.nlm.nih.gov/nuccore/NM_001165960.1 | 36,5 | Medium | 607206 | Predominantly (the highest of two) |
| 5 | **COL17A1**: Homo sapiens collagen type XVII alpha 1 chain (COL17A1), mRNA | https://www.ncbi.nlm.nih.gov/nuccore/NM_000494.3 | 769,3 | Medium/ Low | 113811 | Predominantly |
| 6 | FGFR3: Homo sapiens fibroblast growth factor receptor 3 (FGFR3), transcript variant 3, mRNA | https://www.ncbi.nlm.nih.gov/nuccore/NM_001163213.1 | 333,1 | High | 134934 | Predominantly |
| 7 | TYR: Homo sapiens tyrosinase (TYR), mRNA | https://www.ncbi.nlm.nih.gov/nuccore/NM_000372.4 | 29,8 | High | 604103 | Only |
| 8 | LOR: Homo sapiens loricrin (LOR), mRNA | https://www.ncbi.nlm.nih.gov/nuccore/NM_000427.2 | 532 | Medium/ Low | 606933 | Only |
| 9 | HOXC13: Homo sapiens homeobox C13 (HOXC13), mRNA | https://www.ncbi.nlm.nih.gov/nuccore/NM_017410.2 | 4,6 | Only in hair | 142976 | Only |
| 10 | KRT2: Homo sapiens keratin 2 (KRT2), mRNA | https://www.ncbi.nlm.nih.gov/nuccore/NM_000423.2 | 2600 | High | 600194 | Predominantly (one of three) |

1. **NOT CAUSING DISEASES GENES**

|  | **GENE** | **NCBI LINK** | **RNA TS TPM*** | **PROTEIN EXPRESSION (score)**** | **TISSUE SPECIFICITY** |
| --- | --- | --- | --- | --- | --- |
| **1** | **DCT**: Homo sapiens dopachrome tautomerase (DCT), transcript variant 1, mRNA | https://www.ncbi.nlm.nih.gov/nuccore/NM_001922.4 | 126,8 | Medium/ High | Only |
| **2** | **PMEL**: Homo sapiens premelanosome protein (PMEL), transcript variant 1, mRNA | https://www.ncbi.nlm.nih.gov/nuccore/NM_001200054.1 | 133,4 | Medium/ High | Only |
| **3** | **GSDMA**: Homo sapiens gasdermin A (GSDMA), mRNA | https://www.ncbi.nlm.nih.gov/nuccore/NM_178171.4 | 58,4 | Medium/ High | Only |
| 4 | **KLK5**: Homo sapiens kallikrein related peptidase 5 (KLK5), transcript variant 2, mRNA | https://www.ncbi.nlm.nih.gov/nuccore/NM_001077491.1 | 213,5 | Medium | Only |
| 5 | **DMKN**: Homo sapiens dermokine (DMKN), transcript variant 2, mRNA | https://www.ncbi.nlm.nih.gov/nuccore/NM_033317.4 | 3991,5 | High | Predominantly |
| 6 | DSC1: Homo sapiens desmocollin 1 (DSC1), transcript variant Dsc1a, mRNA | https://www.ncbi.nlm.nih.gov/nuccore/NM_024421.2 | 273 | High | Predominantly (one of three) |
| 7 | **KRT77**: Homo sapiens keratin 77 (KRT77), mRNA | https://www.ncbi.nlm.nih.gov/nuccore/NM_175078.2 | 308,7 | High | Predominantly (the highest of four) |
| 8 | PLA2G4E: Homo sapiens phospholipase A2 group IVE (PLA2G4E), mRNA | https://www.ncbi.nlm.nih.gov/nuccore/NM_001206670.1 | 20,4 | Medium/ High | Predominantly |
| 9 | KRTDAP: Homo sapiens keratinocyte differentiation associated protein (KRTDAP), transcript variant 1, mRNA | https://www.ncbi.nlm.nih.gov/nuccore/NM_207392.2 | 4647,8 | Medium/ High | Predominantly |
| 10 | MLANA: Homo sapiens melan-A (MLANA), mRNA | https://www.ncbi.nlm.nih.gov/nuccore/NM_005511.1 | 50,4 | High | Only |
