# Supplementary material for: Calculating and comparing codon usage values in rare disease genes highlights codon clustering with disease-and tissue- specific hierarchy
Source: PLoS One. 2022 Mar 31;17(3):e0265469. doi: 10.1371/journal.pone.0265469 (PMC8970475; doi:10.1371/journal.pone.0265469)
Supplement: S4 Table — In order to prioritize kidney genes, we selected those with higher expression from the kidney-enriched genes list of the Human Protein Atlas database (https://www.proteinatlas.org/search/tissue_specificity_rna:kidney;Tissue%20enriched+AND+sort_by:tissue+specific+score+AND+show_columns:groupenriched). All data (RNA, TS, TPM, Protein expression scores and Tissue specificity) were also obtained by the Human Protein Atlas database. *RNA TS TPM indicates RNA level reported as mean TPM (transcripts per million), in referred tissue, kidney in this case. **Protein expression scores are based on a best estimate of the "true" protein expression from a knowledge-based annotation in the selected tissue, kidney in this case. ***Tissue specificity is based on data found in the graph called “HPA tissue dataset”, a sub-category of the “RNA sample summary” section in the HPA site, for each gene. The RNA summary section shows normal distribution of individual samples across the datasets of multiple RNA-seq analyses visualized with box plots. “Only” is used for a gene transcript present only in the specific tissue (kidney). “Predominantly” is used when the majority of a gene transcript is present in the specific tissue (kidney). “All” is used for a gene transcript present in all tissues. (DOCX) [file pone.0265469.s006.docx]

**Supplementary TABLE 4: Kidney genes**

1. **DISEASE CAUSING GENES**

|  | **GENE** | **NCBI LINK** | **RNA TS TPM*** | **PROTEIN EXPRESSION (score)**** | **OMIM NUMBER** | **TISSUE SPECIFICITY** |
| --- | --- | --- | --- | --- | --- | --- |
| 1 | **UMOD**: Homo sapiens uromodulin (UMOD), transcript variant 2, mRNA | https://www.ncbi.nlm.nih.gov/nuccore/NM_001008389.2 | 2392 | High | 191845 | Only |
| 2 | **SLC12A1**: Homo sapiens solute carrier family 12 member 1 (SLC12A1), transcript variant 1, mRNA | https://www.ncbi.nlm.nih.gov/nuccore/NM_000338.2 | 780 | High | 600839 | Only |
| 3 | **KCNJ1**: Homo sapiens potassium voltage-gated channel subfamily J member 1 (KCNJ1), transcript variant 1, mRNA | https://www.ncbi.nlm.nih.gov/nuccore/NM_000220.4 | 210,8 | High | 600359 | Only |
| 4 | SLC12A3: Homo sapiens solute carrier family 12 member 3 (SLC12A3), transcript variant 1, mRNA | https://www.ncbi.nlm.nih.gov/nuccore/NM_000339.2 | 109,8 | Medium | 600968 | Only |
| 5 | **NPHS2**: Homo sapiens, stomatin family member, podocin (NPHS2), transcript variant 1, mRNA | https://www.ncbi.nlm.nih.gov/nuccore/NM_014625.3 | 93,6 | High | 604766 | Only |
| 6 | BSND: Homo sapiens barttin CLCNK type accessory beta subunit (BSND), mRNA | https://www.ncbi.nlm.nih.gov/nuccore/NM_057176.2 | 10,2 | High | 606412 | Predominantly (the highest of two) |
| 7 | CLDN16: Homo sapiens claudin 16 (CLDN16), mRNA | https://www.ncbi.nlm.nih.gov/nuccore/NM_006580.3 | 51,7 | Medium | 603959 | Only |
| 8 | PKD1: Homo sapiens polycystin 1, transient receptor potential channel interacting (PKD1), transcript variant 1, mRNA | https://www.ncbi.nlm.nih.gov/nuccore/NM_001009944.2 | 2,3 | High | 601313 | All |
| 9 | PKD2: Homo sapiens polycystin 2, transient receptor potential cation channel (PKD2), mRNA | https://www.ncbi.nlm.nih.gov/nuccore/NM_000297.3 | 54,2 | Medium/Low | 173910 | All |
| 10 | ATP6V0D2: Homo sapiens ATPase H+ transporting V0 subunit d2 (ATP6V0D2), mRNA | https://www.ncbi.nlm.nih.gov/nuccore/NM_152565.1 | 59,4 | Medium | 618072 | Only |

1. **NON- DISEASES CAUSING GENES**

|  | **GENE** | **NCBI LINK** | **RNA TS TPM*** | **PROTEIN EXPRESSION (score)**** | **TISSUE SPECIFICITY** |
| --- | --- | --- | --- | --- | --- |
| **1** | **BBOX1**: Homo sapiens gamma-butyrobetaine hydroxylase 1 (BBOX1), mRNA | https://www.ncbi.nlm.nih.gov/nuccore/NM_003986.2 | 473 | High | Predominantly (the highest of two) |
| **2** | **SLC22A8**: Homo sapiens solute carrier family 22 member 8 (SLC22A8), transcript variant 2, mRNA | https://www.ncbi.nlm.nih.gov/nuccore/NM_001184732.1 | 339,8 | Medium | Only |
| **3** | **MIOX**: Homo sapiens myo-inositol oxygenase (MIOX), mRNA | https://www.ncbi.nlm.nih.gov/nuccore/NM_017584.5 | 821,5 | Medium | Only |
| 4 | **TMEM52B**: Homo sapiens transmembrane protein 52B (TMEM52B), transcript variant 2, mRNA | https://www.ncbi.nlm.nih.gov/nuccore/NM_001079815.1 | 183,5 | Medium | Predominantly (the highest of two) |
| 5 | **TINAG**: Homo sapiens tubulointerstitial nephritis antigen (TINAG), mRNA | https://www.ncbi.nlm.nih.gov/nuccore/NM_014464.3 | 113,9 | Low | Predominantly (one of two) |
| 6 | CALB1: Homo sapiens calbindin 1 (CALB1), mRNA | https://www.ncbi.nlm.nih.gov/nuccore/NM_004929.3 | 336,9 | High | Predominantly (one of two) |
| 7 | **ATP6V1G3**: Homo sapiens ATPase H+ transporting V1 subunit G3 (ATP6V1G3), transcript variant 3, mRNA | https://www.ncbi.nlm.nih.gov/nuccore/NM_001320218.1 | 21,3 | Medium | Only |
| 8 | AQP6: Homo sapiens aquaporin 6 (AQP6), mRNA | https://www.ncbi.nlm.nih.gov/nuccore/NM_001652.3 | 20,8 | High | Only |
| 9 | FXYD4: Homo sapiens FXYD domain containing ion transport regulator 4 (FXYD4), transcript variant 1, mRNA | https://www.ncbi.nlm.nih.gov/nuccore/NM_173160.2 | 127,4 | Medium | Only |
| 10 | GGACT: Homo sapiens gamma-glutamylamine cyclotransferase (GGACT), transcript variant 1, mRNA | https://www.ncbi.nlm.nih.gov/nuccore/NM_033110.2 | 75,9 | Low | Only |
