# Supplementary material for: Calculating and comparing codon usage values in rare disease genes highlights codon clustering with disease-and tissue- specific hierarchy
Source: PLoS One. 2022 Mar 31;17(3):e0265469. doi: 10.1371/journal.pone.0265469 (PMC8970475; doi:10.1371/journal.pone.0265469)
Supplement: Attachment — Submitted filename: Answers ref 21.1.22.docx [file pone.0265469.s015.docx]

We thank the referee for the useful comments and here below we provided our replies:

**In response to Referee 1**:
1.Line 65, “genes casing human rare diseases”: Amended in the manuscript text
2. Line 93 “an extreme CUB was identified in both human and chimpanzee compared to other mammals, suggesting its evolutionary meaning in processes plays an exclusive role in a specific eukaryotic lineage” : This part could be rephrased making it clearer: Amended in the manuscript text
3. Concerning this part :
95 “A large consensus exists about the concept that the choice of a synonymous codon does affect… for all genes [8]”. - It is an important statement that is being made in the manuscript, about the consensus that exists around “choice of a synonymous codon does affect protein translation efficiency, expression level, structure, and function, a notion that has prompted”.-This statement, is not adequately represented by references, hence some more references would further develop it. We added more reference to support the importance of the statement we reported in the manuscript.

4. -At the results section “CUB fingerprint across mammals” it is stated from line 277 until line 284 that“…no clear tissue behavior or even specific gene-related clustering can be observed. This finding further supports that CU values show an mammals-related but with important gene-specific differences, which contribute to generate the CUB fingerprints”
Although this statement can stand on its own, if it could be commented along with the statement at lines 92-94 concerning chimps and humans…
92- “an extreme CUB was identified in both human and chimpanzee compared to other 93mammals, suggesting its evolutionary meaning in processes plays an exclusive role in a 94specific eukaryotic lineage” Amended in the manuscript text

5. In the discussion part, at lines 403 to 404, where a hypothesis is presented concerning the reason of CUB , it is stated that positive selection of specific codons could putatively be the reason for this fact. Since it is an aspect of the “story” the manuscript unfolds, maybe these codons should be mentioned along with a more specific hypothesis, as it is very nicely done at the following lines concerning muscle tissue and the stop codon. Amended in the manuscript text
**In response to Referee 2**

1-Authors only focused on tissue-specific genes. I'm wondering whether authors also calculated CU values in housekeeping genes related to disease ( disease-causing genes) vs genes not related to disease

We apologies to the reviewer for having not explained enough clearly our approach. Our rationale was to consider tissue-specific genes only, either for non-disease causing (“control”) and disease causing genes and to compare them. Therefore, in this proof-of-principle study, we did not include housekeeping genes. Indeed, we definitively believe that housekeeping genes, almost ubiquitously expressed, would deserve to be studied by the same approach. This is something we will certainly do in further studies. In this paper we were focused on tissue specificity, since having genes with same expression site/level facilitated CU values calculation and comparison between gene groups.

Why did authors analyze CU values in 15 mammalian species ? Do these species develop the same diseases ? What is the scientific background regarding it ?

Thanks for this question which allows to clarify the evolutionary analyses. In order to make more robust the identification of rare codons, we checked conservation of selected genes (both disease and non-disease causing) across mammals. We limited our selection to mammals to avoid to get i) too many bias due to the large specie variability, ii) to compare homo sapiens genes to species evolutionarily close (mammals and non-human primates). Regarding disease propensity, we disregarded wether species may develop human-like diseases, for two main reasons: this occurs very rarely (the so called natural animal disease models), and we were interested in comparing human genes only, since certainly related to diseases. Indeed, CUB was conserved among species, with however some difference in the heatmaps observed in disease-causing genes, especially those related to muscle diseases.

Concluding, analyzing CU values across mammals provided a further support of the different CUB behavior (we found conserved among species) between disease and non-disease genes. We have modified and added a sentence (Introduction) to clarify this concept.
